# Effects of the micronutrient Sanopal® (5-hydroxymethyl-2-furfural and α-ketoglutaric acid) on oxygen affinity of hemoglobin, oxygen saturation and exercise responses at altitude

**DOI:** 10.1080/15502783.2026.2643684

**Published:** 2026-03-17

**Authors:** Simon Woyke, Teresa Troppmair, Norbert Mair, Herbert Oberacher, Thomas Haller, Martin Faulhaber, Hannes Gatterer

**Affiliations:** a Department of Anaesthesiology and Intensive Care Medicine, Medical University of Innsbruck, Innsbruck, Austria; b Department of Physiology and Medical Physics, Institute of Physiology, Medical University of Innsbruck, Innsbruck, Austria; c Institute of Legal Medicine and Core Facility Metabolomics, Medical University of Innsbruck, Innsbruck, Austria; d Department of Sport Science, University of Innsbruck, Innsbruck, Austria; e Institute of Mountain Emergency Medicine, Eurac Research, Bolzano, Italy

**Keywords:** Hemoglobin, nutritional supplement, 5HMF, P50, oxygen affinity

## Abstract

**Background:**

With increasing altitude, the partial pressure of oxygen and oxygen saturation (SpO_2_) decrease, reducing physical performance. This study investigates whether the nutritional supplement Sanopal® (5-hydroxymethyl-2-furfural and *α*-ketoglutaric acid) increases hemoglobin-oxygen affinity and SpO₂ during exercise at moderate altitude.

**Methods:**

Nineteen healthy young sports students (12 females, 7 males) participated in a single-blinded, placebo-controlled crossover study investigating the effects of Sanopal® at low (590 m) and moderate altitude (2900 m). Participants received Sanopal® or placebo in a randomized order, with measurements of SpO₂, heart rate, and blood parameters taken before and after ingestion, as well as before and after exercise at altitude.

**Results:**

Under resting and acute hypoxia conditions, Sanopal® did not increase hemoglobin-oxygen affinity or SpO₂. At altitude and post-exercise, Hb-O₂ affinity decreased by approximately 5% in the PL trial but increased by approximately 2% in the SA session (interaction effect: *p* = 0.030). There were no significant differences in SpO₂ or heart rate between the Sanopal® and placebo groups.

**Conclusions:**

Sanopal® did not alter hemoglobin-oxygen affinity or SpO₂ under resting conditions in normoxia or acute hypoxia. After exercise at altitude, it slightly increased Hb-O₂ affinity without significantly affecting SpO₂ or other measured blood parameters. The modest increase in Hb-O_2_ affinity following exercise may have limited the exercise-induced decrease in Hb-O_2_ affinity. However, this increase was likely too small to significantly raise SpO₂ in this cohort at a relatively low altitude.

## 1. Introduction

As altitude increases, barometric pressure decreases, resulting in a lower inspiratory partial pressure of oxygen. This subsequently reduces alveolar and arterial oxygen partial pressure (PaO₂) and arterial oxygen saturation (SaO₂) [1]. This decrease triggers a series of acute physiological responses aimed at restoring oxygen delivery to the tissues, e.g. increased ventilation and cardiac output [2–5]. Additionally, hemoconcentration is initiated to increase the hemoglobin concentration and thereby arterial oxygen content [6,7]. A factor often ignored in this regard is the binding capacity of oxygen to hemoglobin, described by the oxygen dissociation curve (ODC) [8]. Since reductions in SpO_2_ are related to decrements in endurance performance at altitude [9], it may be inferred that a shift of the ODC to the left, which increases SpO_2_, may also influence exercise performance [10]. Obviously, it needs to be considered that a left-shift of the ODC might impair peripheral O_2_ unloading. However, recent data on hypoxic exercise shows that a high O_2_ affinity of hemoglobin (Hb-O_2_), and hence enhanced oxygen uptake in the lungs, outweighed deficits in peripheral O_2_ unloading [11]. Additionally, it was reported previously that under hypoxic conditions, O_2_ unloading from Hb does not require a right-shift of the ODC [12].

Various physiological parameters affect the ODC (e.g. 2,3-bisphosphoglycerate, temperature, partial pressure of carbon dioxide, pH, electrolytes, etc.) [13–15], while some are still obscure [16]. There are additional substances that are able to shift the ODC to the left. One of those is 5-hydroxymethyl-2-furfural (5-HMF) – a nutritional supplement. 5-HMF reduces the P50 (PaO_2_ at which 50% of hemoglobin is saturated with oxygen, a parameter indicating the position of the ODC) via allosteric modification of hemoglobin by creating a Schiff-base adduct [17]. In animal studies, 5-HMF improved SaO_2_ and preserved systemic oxygen delivery in hypoxia [17,18]. 5-HMF was also found to increase the oxygen affinity in healthy subjects exposed to hypoxia [19]. Recently, we found that a commercially available supplement (i.e. Sanopal®), which, in addition to 5-HMF also includes *α*-ketoglutaric acid (αKG), increased SpO_2_ during cycling exercise at 3500 m in healthy subjects [20], the underlying mechanism might be an increase in Hb-O_2_ affinity. Moreover, in an in vitro experiment, we observed dose-dependent and sex-specific modifications of the Hb-O_2_ affinity with this supplement [21]. The aim of this study was to investigate whether the in vitro results could be replicated in vivo by increasing Hb-O_2_ affinity at rest and during submaximal exercise at a real altitude of 2900 m. We hypothesized that Sanopal® (SA) ingestion (a) increases oxygen affinity measured from capillary blood under resting and submaximal exercise conditions at sea level and at altitude and (b) increases SpO_2_ at rest and during submaximal exercise at altitude.

## 2. Methods

### 2.1. Study participants and pre-screening procedures

Twenty-two young (aged 18–35 years), healthy female and male sports students of the Department of Sport Science (University of Innsbruck, Austria) volunteered to participate in the study. Three of the 22 participants were unable to complete the trials due to illness developing on the second experimental day (*n* = 2), or because of missing measurements (*n* = 1). The final sample therefore included 19 participants, 12 females and 7 males with a median age of 21 (IQR 20–22) years. The study was conducted in accordance with the Declaration of Helsinki and was approved by the Board for Ethical Questions in Science of the University of Innsbruck (nr. 107/2023). Written informed consent was given by all participants.

Prior to inclusion in the study, participants underwent a routine screening (adapted Physical Activity Readiness Questionnaire – PAR-Q). If there were any abnormalities in the information provided in the questionnaire, a physician was consulted for further clarification. The exclusion criteria were acute or chronic diseases, pregnancy (verified by a commercially available pregnancy test), regular cigarette smoking (more than 5 cigarettes per day), recent blood loss through trauma or surgery (last 2 weeks), living at an altitude above 1500 m, as well as a stay at altitudes above 2500 m in the last 2 weeks before the start of the study and during the duration of the study.

### 2.2. Procedures

The study was designed as a single-blinded, placebo-controlled, cross-over trial. The participants presented to the laboratory at the Department of Sport Science (University of Innsbruck) twice (at least one week apart), once for the placebo (PL) and once for the verum session (SA). The order was randomly assigned. Each session included 2 resting measurements at low altitude (590 m) followed by 1 resting and 1 exercise measurement at moderate altitude (2900 m).

The supplement Sanopal® (SA), which was administered in this study, contains 5-HMF and aKG and is a commercially available nutritional supplement used during surgery, competitive sports, and other physical or mental abnormal situations (e.g. burnout, depression). SA was administered in a triple dose, split into a double dose (ingested immediately after the baseline measurements) and a single dose (ingested before cable car transportation to altitude). A triple dose is considered safe according to the manufacturer.

On arrival at the laboratory (590 m), the first resting measurements, including SpO_2_ and heart rate (HR) (NONIN WristOx2® Model 3150, Medical, Plymouth, MN, USA), were taken after 5 min of rest in a sitting position. Afterwards, capillary blood from the hyperemized earlobe was collected. Subsequently, the participants took a double dose of SA dissolved in 300 ml of water or a placebo drink (similar in color and taste, containing mainly carbohydrates). After 45 min, the second resting measurements, including SpO_2_, HR, and capillary blood sampling, were conducted. Next, the participants were transported to the valley station of the Stubaier Glacier cable car (1750 m; Stubaital, Tyrol) by car (approximately a 1-h drive), where a further single SA or placebo dose was ingested. Then, the participants took the cable car to the top station (2900 m), where 45 min after arrival, the third resting measurements were taken (SpO_2_, HR, and capillary blood sampling). Finally, a step test was performed in a slightly modified form as suggested by [22] (5 min instead of 2 min), and SpO_2_ spot measurement was taken during the last seconds of the exercise test. This was then followed by the last capillary blood collection (exercise measurements).

The ODC was measured in vitro by a high-throughput method designed for complete curve recordings in untreated whole blood samples at all time points [23]. 2,3-BPG concentrations were determined using liquid chromatography–tandem mass spectrometry from blood taken 45 min after arrival at altitude [24,25]. The base excess (BE) and lactate concentration ([La]) were determined using a blood gas analyzer (Siemens Rapidpoint 500e) on blood samples taken after exercise at altitude.

### 2.3. Statistics

The data were analyzed with IBM SPSS Statistics (version 26). A normal distribution was confirmed by using the Shapiro‒Wilk test, and all variables except SpO_2_ at some time points, were normally distributed. A 2×3 ANOVA repeated measurement design with 2 within-subject factors (condition and time point of measurement) and including sex as a between-subject factor, was used to investigate differences in the resting parameters of P50, the Hill coefficient (HC), SpO_2_, and HR between interventions (i.e. PL vs. SA) at three different time points (i.e. baseline, 45 min after ingestion and 45 min after arrival at altitude). Furthermore, a 2×2 ANOVA was run for the same parameters before and after exercise (i.e. baseline and exercise) and between intervention (i.e. PL vs SA). ANOVA was also used for SpO₂, even though it was not normally distributed. The literature shows that ANOVA with repeated measurements is largely robust to violations of the normal distribution assumption, provided that this is the only assumption that has been violated [26]. If 2×3 ANOVA detected differences, LSD post hoc tests were applied to locate those. Paired sample *t*-tests were used to compare differences in [La] and BE after exercise at altitude, as well as resting 2,3-BPG at altitude, between interventions. Owing to insufficient blood volume, values for 2,3-BPG, [La], and BE were only available for a limited number of participants (*n* = 6, *n* = 16, and *n* = 16, respectively). The data are presented as means ± SD. In addition to the *p*-values, the partial eta-squared (η²) effect size for the ANOVA results is shown, with small, medium, and large effects represented by values of 0.01, 0.06, and 0.14, respectively [27]. The level of significance was set at *p* < 0.05. Since our in vitro experiments showed a clear reduction in P50 by the supplement [21], one-sited testing was applied for this parameter.

## 3. Results

**Figure 1.**
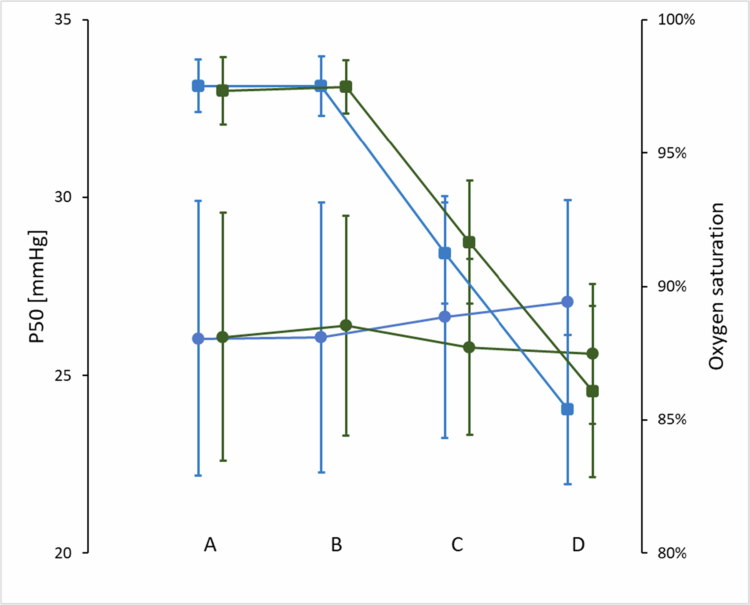
P50 (circles, left y-axis) and SpO_2_ (squares, right y-axis) at time points: A (before Sanopal/placebo ingestion in normoxia), B (45 min after Sanopal/placebo ingestion in normoxia), C (at arrival at altitude), and D (immediately after exercise at altitude) for Sanopal (green) and placebo (blue).

Under resting conditions in normoxia or acute hypoxia, SA did not increase Hb-O_2_ affinity or SpO_2_ (Table 1). After exercise, an interaction effect (intervention × time) was found for P50 only (Table 2). Overall, no interaction effects for sexes (time point × sex and intervention × sex) were detected (*p* > 0.226), except for HC under resting conditions (time point × sex, *p* = 0.049). Compared to near sea level, the resting SpO_2_ was lower at altitude in all participants, and decreased further immediately after exercise (Tables 1 and 2). The heart rate remained unchanged at altitude but was significantly higher after exercise (Tables 1 and 2). There was no difference in SpO_2_ or heart rate between the SA and the PL trial. Figure 1 shows the overall time course of P50 and SpO2 changes for the SA and PL settings.

No effect of the intervention was observed for HC (see Tables 1 and 2). In a limited number of participants, no differences in 2,3-BPG were detected 45 min after arrival at altitude between SA and PL (12.5±2.5 µmol/gHb for SA vs. 12.8±1.8 µmol/gHb for PL, *p* = 0.796). [La] and BE immediately after exercise at altitude were not statistically significant different between the trials ([La]: 3.3±0.8 vs. 3.8±1.3 mmol/L, for SA and PL, respectively, *p* = 0.188; BE: −3.6±1.7 vs. −4.1±2.1, for SA and PL, respectively, *p* = 0.497).

**Table 1.** Resting data at baseline, 45 min after the first dose at low altitude (Post LA) and 45 min after arrival at moderate altitude (post MA) for the Sanopal® (SA) and the placebo (PL) session.

				ANOVA		
	Baseline	Post LA	Post MA	main effect intervention *p*-value (ES)	Main effect time *p*-value (ES)	Interaction effect (intervention x time) *p*-value (ES)
P50 SA [mmHg]	26.1±3.6	26.5±3.1	25.9±2.5	0.471 (<0.001)	0.524 (0.037)	0.227 (0.044)
P50 PL [mmHg]	26.0±3.7	26.4±3.5	26.8±3.3			
HC SA	2.50±0.26	2.49±0.23	2.57±0.30	0.969 (<0.001)	0.212 (0.088)	0.275 (0.073)
HC PL	2.51±0.31	2.54±0.19	2.53±0.29			
SpO_2_ SA [%]	97.4±1.3	97.4±1.0	91.8±2.3	0.670 (0.011)	<0.001 (0.919)	0.447 (0.042)
SpO_2_ PL [%]	97.6±1.0	97.5±1.2	91.3±2.0			
Heart rate SA	75±8	71±8	70±11	0.604 (0.016)	<0.001 (0.528)	0.243 (0.080)
Heart rate PL	77±11	69±8	68±10			

Effect size (ES): partial η².

**Table 2.** Changes in values from baseline to post-exercise at altitude for Sanopal® (SA) vs. placebo (PL).

			ANOVA		
	*SA*	PL	main effect intervention *p*-value (ES)	Main effect time *p*-value (ES)	Interaction effect (intervention x time) *p*-value (ES)
∆P50 [mmHg]	−0.50 ± 3.10	1.25 ± 3.22	0.153 (0.065)	0.377 (0.049)	0.030 (0.202)
HC	−0.05 ± 0.19	0.06 ± 0.37	0.457 (0.035)	0.724 (0.008)	0.113 (0.150)
SpO_2_ [%]	−11.3 ± 3.5	−12.1 ± 2.6	0.750 (0.007)	<0.001 (0.959)	0.604 (0.017)
Heart rate [bpm]	47 ± 21	51 ± 17	0.543 (0.024)	<0.001 (0.900)	0.789 (0.005)

Effect size (ES): partial η².

## 4. Discussion

The main findings of this study are that, under resting conditions in both normoxia and acute hypoxia, SA does not alter Hb-O₂ affinity or SpO₂ compared to PL. However, immediately following exercise, SA increased Hb-O₂ affinity without affecting SpO₂. No significant differences were observed between trials in BE, [La], or 2,3-BPG levels.

From a physiological perspective, high-altitude exposure leads to an increased Hb-O_2_ affinity, primarily due to respiratory alkalosis induced by hyperventilation. At moderate altitudes, this effect is largely offset by an alkalosis-driven increase in 2,3-bisphosphoglycerate (2,3-BPG), which shifts the ODC back toward sea-level norms [25]. However, at very high or extreme altitudes, the extent of respiratory alkalosis surpasses the compensatory rise in 2,3-BPG, resulting in a net increase in Hb-O₂ affinity [28]. Shifts in the ODC influence SpO_2_ levels at altitude. Elevations in SpO_2_ levels, presumably through increased Hb-O_2_ affinity, are thought to improve exercise capacity at altitude [10,20].

Although we previously reported that the combination of 5-HMF and αKG strongly affects Hb-O₂ affinity in vitro in a sex- and dose-dependent manner [21], we did not observe any such effects under resting conditions in normoxia or acute hypoxia in the present study. As plasma concentrations of SA were not measured, we cannot confirm whether effective systemic exposure was achieved. Most likely, the effective systemic exposure of hemoglobin to 5-HMF and αKG was lower in vivo than in vitro due to limited biological availability. Nonetheless, at altitude and after exercise, in the PL trial, Hb-O_2_ affinity decreased by approximately 5%, while during the SA session, Hb-O_2_ affinity slightly increased by approximately 2% at the same time. Although there were no statistically significant changes in BE and [La] between the interventions after exercise, SA ingestion might have eliminated or limited the effect of exercise on Hb-O_2_ affinity (i.e. reduction of Hb-O_2_ affinity by the Bohr effect). 2,3-BPG levels were not increased at altitude, probably because of the relatively short time at moderate altitude (45 min) before blood sampling [25,29] or the very limited sample size. Additionally, previous studies have shown that acute exercise does not lead to decreased 2,3-BPG levels as long as lactic acidosis is not severe [30], while performing exercise on a daily basis supports the increase of 2,3-BPG levels at moderate altitude [31].

SpO_2_ did not increase in the SA session compared to the PL session in this study. We assume that the small yet statistically significant increase in Hb-O_2_ affinity with SA supplementation after exercise at altitude was not large enough to cause a significant increase in SpO_2_ in this rather small cohort. While 5-HMF increases Hb-O_2_ affinity by binding to hemoglobin, at high altitudes, this effect might be further enhanced by altitude-induced respiratory alkalosis. If the combination of these two effectors is synergistic, additive or attenuated effects is unknown. During exercise, especially in hypoxic conditions, the ODC can be affected by changes in pH, PCO_2_ and increases in temperature, particularly at the tissue level in the working skeletal muscle [12]. While the effects of pH, PCO_2_, and temperature are dynamically regulated and short-lived, the 5-HMF effect should last for as long as the substance remains available. The complex interaction of several effectors with different dynamics is difficult to assess, yet it influences Hb-O_2_ affinity. In contrast to the present study, Kossler et al. [20] reported a significant increase in SpO₂ in eight moderately trained male volunteers during a 2-h cycle time trial in hypoxia [20]. The severity of hypoxia (simulated altitude of 3500 m) and exercise intensity were greater in their study, leading to overall lower SpO₂ values compared to the present investigation. In the present investigation, SpO_2_ levels at altitude were approximately 90%. At this point, the ODC turns into its asymptotic range, i.e. changes in PO_2_ affect SO_2_ only to a small degree, limiting its sensitivity. Furthermore, we applied spot SpO_2_ measurements instead of continuous measurements, potentially missing differences in the time course during the altitude stay. We assume these are the reasons why we might have missed a statistically significant increase in SpO_2_ in our study.

Further studies are needed to investigate systemic SA bioavailability, metabolism, and adequate dosage use to ensure a detectable and relevant effect on Hb-O_2_ affinity and potentially SpO_2_. Furthermore, it needs to be addressed whether such changes may affect exercise performance at altitude.

### 4.1. Limitations

As mentioned earlier, plasma levels of 5-HMF and αKG have not been measured; therefore, the systemic bioavailability of SA ingestion, and the concentrations of 5-HMF and αKG to which red blood cells are exposed, are unknown. Furthermore, spot measurements of SpO₂ might not adequately reflect SpO₂ values over the course of the altitude stay. Finally, missing values for some parameters may limit the validity of these results. Participants’ physical activity and dietary intake, which both might be confounding factors, were not recorded or standardized in this study.

## 5. Conclusion

In conclusion, SA ingestion does not increase Hb-O_2_ affinity at rest but may prevent a right-shift of the ODC during exercise at moderate altitude. Since we were not able to detect an effect on SpO_2_, potentially owing to the relatively low altitude, the effects of the changed Hb-O_2_ affinity on exercise responses and, ultimately, performance should be addressed in future studies.

## Data Availability

The data that support the findings of this study are available from the corresponding author upon reasonable request.
